# Biomechanic characterization of normal urethra using uro-dynamic MRI during voiding

**DOI:** 10.1007/s11255-026-05069-5

**Published:** 2026-03-23

**Authors:** Cody Johnson, Juan Pablo Gonzalez-Pereira, Maxwell Kounga, Wade Bushman, Alejandro Roldan-Alzate

**Affiliations:** 1 Department of Radiology, University of Wisconsin-Madison, Madison, USA; 2 Department of Mechanical Engineering, University of Wisconsin-Madison, Madison, USA; 3 Department of Urology, University of Wisconsin-Madison, Madison, USA; 4 Department of Mechanical Engineering, Department of Radiology, Department of Biomedical Engineering, Department of Urology, University of Wisconsin-Madison, Madison, USA

**Keywords:** Urethral biomechanics, Dynamic MRI, Anterior urethra, Posterior urethra, Urethral resistance, Urethral diameter

## Abstract

**Introduction:**

Dynamic volumetric MRI was used to non-invasively assess urethral biomechanics in a single healthy male subject during voiding.

**Methods:**

Volumetric lower urinary tract (LUT) images were obtained throughout the voiding effort using the uro-dynamic MRI methodology. These were subsequently segmented using MIMICS. Segmented urethral volumes were divided into prostatic, membranous, penile and bulbous urethra and were used to quantify urethral diameters over time, length and different phases of flow. Idealized instantaneous resistances were calculated for the posterior (prostatic and membranous) urethra.

**Results:**

The analysis was separated into time and length averaged, and step-by-step analysis. Step-by-step analysis revealed more variation in the initial flow and terminal flow than during robust flow. Time and length averaged analysis showed the membranous urethra had the narrowest lumen and greatest resistance to flow. Correlation of urethral diameters with flow showed strong correlations with both the prostatic and membranous urethral segments.

**Conclusion:**

This single-subject study confirms the potential of uro-dynamic MRI to provide noninvasive assessment of lower urinary tract anatomy and urethral biomechanics during voiding.

## Introduction

Magnetic resonance imaging (MRI) has proven to be a useful method to analyze lower urinary tract anatomy and function, as it is non-invasive, accurate, and easily repeatable [1–4]. These are significant advantages over current diagnostic methodologies, including cystoscopy, retrograde urethrography, and multichannel urodynamics with fluoroscopic imaging (video-urodynamics or UDS), as they are invasive and provide limited information [5–7]. Recent advancements in MRI have allowed for dynamic imaging of a voiding event. Previous studies have used uro-dynamic MRI technique to image the bladder and prostate during voiding [8–12]. We report here a single-subject study expanding these imaging techniques [10–12] to the urethra and describe the dynamic changes in urethral anatomy and diameter during voiding.

**Fig. 1:**
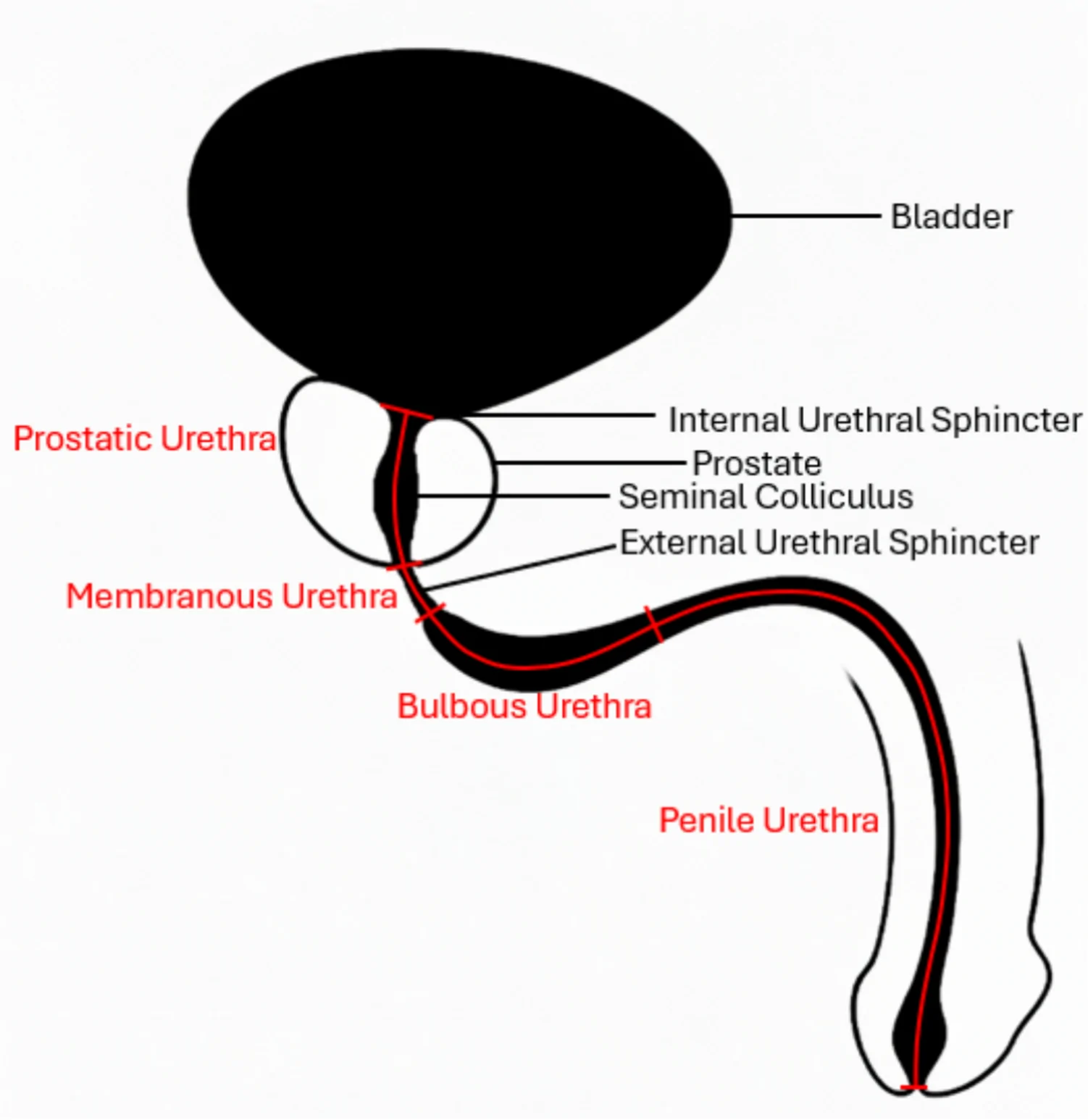
An image displaying the anatomy of the lower urinary tract, with the urethra sub-divided into 4 urethral segments (prostatic, membranous, bulbous, and penile)

The urethra serves as the conduit for voiding urine from the bladder. The posterior urethra includes the prostatic and membranous urethra, while the anterior urethra is comprised of the bulbous and penile urethra (Fig. 1). The effects of prostatic obstruction and anterior urethral strictures on voiding have received considerable attention and study [13–18], but the membranous urethra remains relatively understudied. The membranous urethra is an urothelium-lined tube surrounded by a sleeve muscle; it is bordered by the prostatic urethra and bulbous urethra and traverses the striated muscular pelvic diaphragm. Together, the membranous urethra and pelvic diaphragm comprise the external urinary sphincter. The membranous urethra is closed except during voiding and plays a major role in maintaining continence. The membranous urethra opening during voiding is considered an active process of relaxation rather than a result of urine voiding pressure [19]. Functional urodynamic studies complemented by fluoroscopy have shown that even while relaxed and open, the membranous urethra is normally the point of maximal resistance in the outflow tract [20, 21]. This study aims to implement dynamic MRI techniques, previously used for the characterization of bladder biomechanics, to the urethra. The focus of this novel technology is to quantitatively characterize dynamic morphological changes in the posterior urethra during voiding which will help addressing a gap in the functional understanding of this urethral region.

## Materials and methods

In this HIPAA compliant, IRB approved study (University of Wisconsin-Madison IRB-approved study (2021-1247), Ethical Approval Number (2021-1247)), one 29-year-old healthy male with an IPSS score less than 7 and no contraindications to MRI, was recruited to void supine in an MRI scanner to acquire dynamic images of the voiding event.

### MRI acquisition

Scanning was performed on a clinical 3T scanner (Premier, GE Healthcare, Waukesha, WI) using a high-density flexible surface coil array (AIR coil, GE Healthcare). The acquisition sequence used was 3D differential subsampling with cartesian ordering (DISCO) flex (Spatial resolution: 1 mm isotropic; slice thickness: 2 mm) which acquired dynamic imaging of the urethra throughout the void. The subject hydrated prior to the study by generous water intake both at home and at site. 15 min prior to the scans, 1/3 of a single weighted-base dose (0.1 mmol/kg) of gadolinium-based contrast was slowly injected intravenously and a condom catheter was applied. To ensure proper image quality and contrast mixing an initial mask is taken before the voiding sequence. If there is improper mixing the subject is instructed to roll on their side for 4 s intervals to ensure proper mixing. This process is repeated until contrast is fully mixed. Once the DISCO flex acquisition was initiated, the patient was instructed to void, and the dynamic imaging of the urethra was collected every 3.82 s throughout the void. These images, in DICOM (Digital Imaging and Communications in Medicine) format, were uploaded into the software Mimics (Materialise, Leuven, Belgium; Version 23) for analysis of urethral anatomy, function, and biomechanics.

### Image processing

**Fig. 2:**
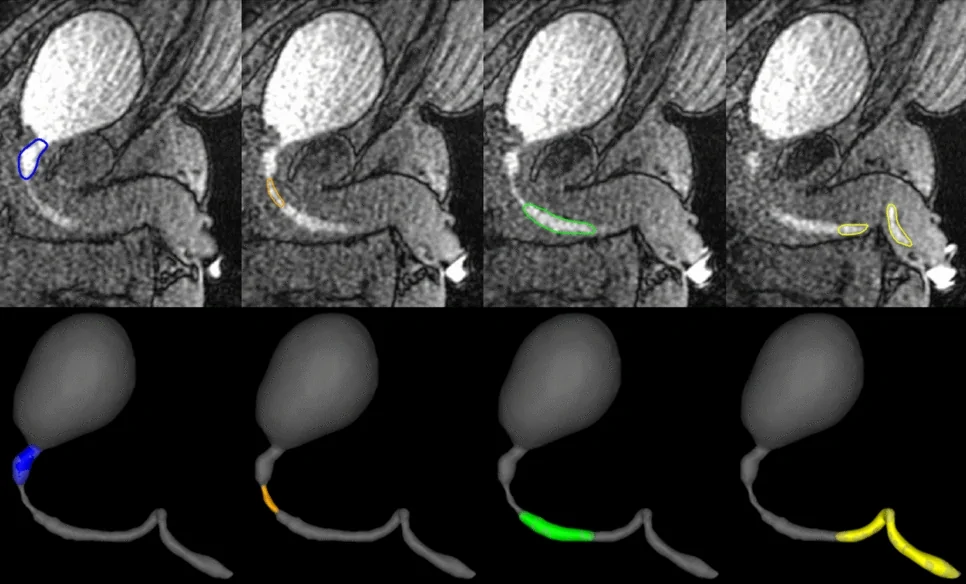
**a** Contour lines of the prostatic (blue), membranous (orange), bulbous (green), and penile (yellow) urethras’ segmentations overlayed on a DISCO flex oblique sagittal MR image at maximum flow rate (42.02 s). **b** 3D anatomical renderings of the urethral subdivisions produced from segmentations of images obtained at maximum flow. The internal urethral sphincter (IUS) located at the junction of the bladder and prostate is not specifically indicated. Note that the apparent kink in the penile urethra is due to an axial curvature and is not a luminal narrowing

Urine flow occurred for 95.5 s. This event was subdivided into twenty-five 3.82-s time steps for analysis with image segmentation performed at each time interval in Mimics. All image segmentations used semi-automatic tools with manual corrections for errors and image artifact. For purposes of anatomic correlation, the urethra was manually subdivided into prostatic, membranous, bulbous, and penile segments based on anatomic location and were verified by an urologist (Fig. 2a). The prostatic urethra is contained completely within the prostate, the membranous urethra begins at the end of the prostate and traverses the pelvic diaphragm, the bulbous urethra begins at the external sphincter and ends at the penoscrotal junction. The product of these segmentations and subdivisions was the creation of 3D renderings of the full length of the urethra and its subdivisions (Fig. 2b). Segmentation variability on the 3D renderings might be affected by artifacts generated from bladder wall motion. Inter and intra reader variability studies are commonly used to evaluate the effects of these sources on segmentations form medical imaging. Segmentation reproducibility was evaluated by using segmentations performed by two trained scientists to calculate inter and intra-reader variability. All segmentations were verified by an urologist and a radiologist.

### Data collection and calculations

**Fig. 3:**
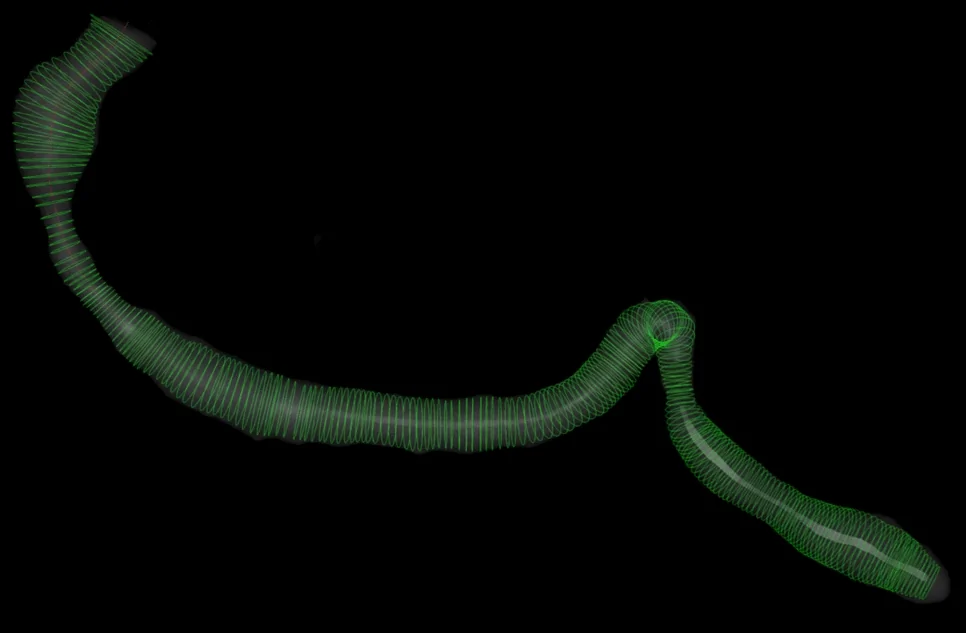
3D rending of the urethra at the maximum flow rate (42.02 s), with best fitted diameters in green across the length of the urethra. The diameters are automatically generated and suitable to be exported

Using the 3D urethra renderings created for each individual time step, and the “Centerline” and “Best Fitted Diameter” tools in Mimics, diameter measurements were able to be obtained at any point along the length of the urethra (Fig. 3). These diameter measurements were exported along the length of the urethra at about every 2 mm for each of the 25-time steps. Additionally, the length of the urethra and the length of urethral subdivisions were also obtained using the “Centerline” tool.

Flow rate and urine flow curve were calculated from changes in bladder volume as described by the uro-dynamic MRI methodology [10]. Additionally, because urine is mostly water and is considered a Newtonian fluid with stable density and viscosity at body temperature an idealized instantaneous resistance was calculated as a function of viscosity, length, and radius for each posterior urethral segment, at every time point, using a derivation from the Hagen-Poiseuille equation (Eq. 1). This derivation assumes rigid tube approximations and steady flow conditions for each urethral segment at each time point. Segment length was obtained from the centerline measurement, urethral radius was calculated from the best fitted diameters, and quantities for urine density and viscosity were 1.035 g/cm^3^ and 0.8293 mPa*s or cP, respectively [22].

1 $$R= \frac{8\eta L}{\pi {r}^{4}}$$

## Results

**Fig. 4:**
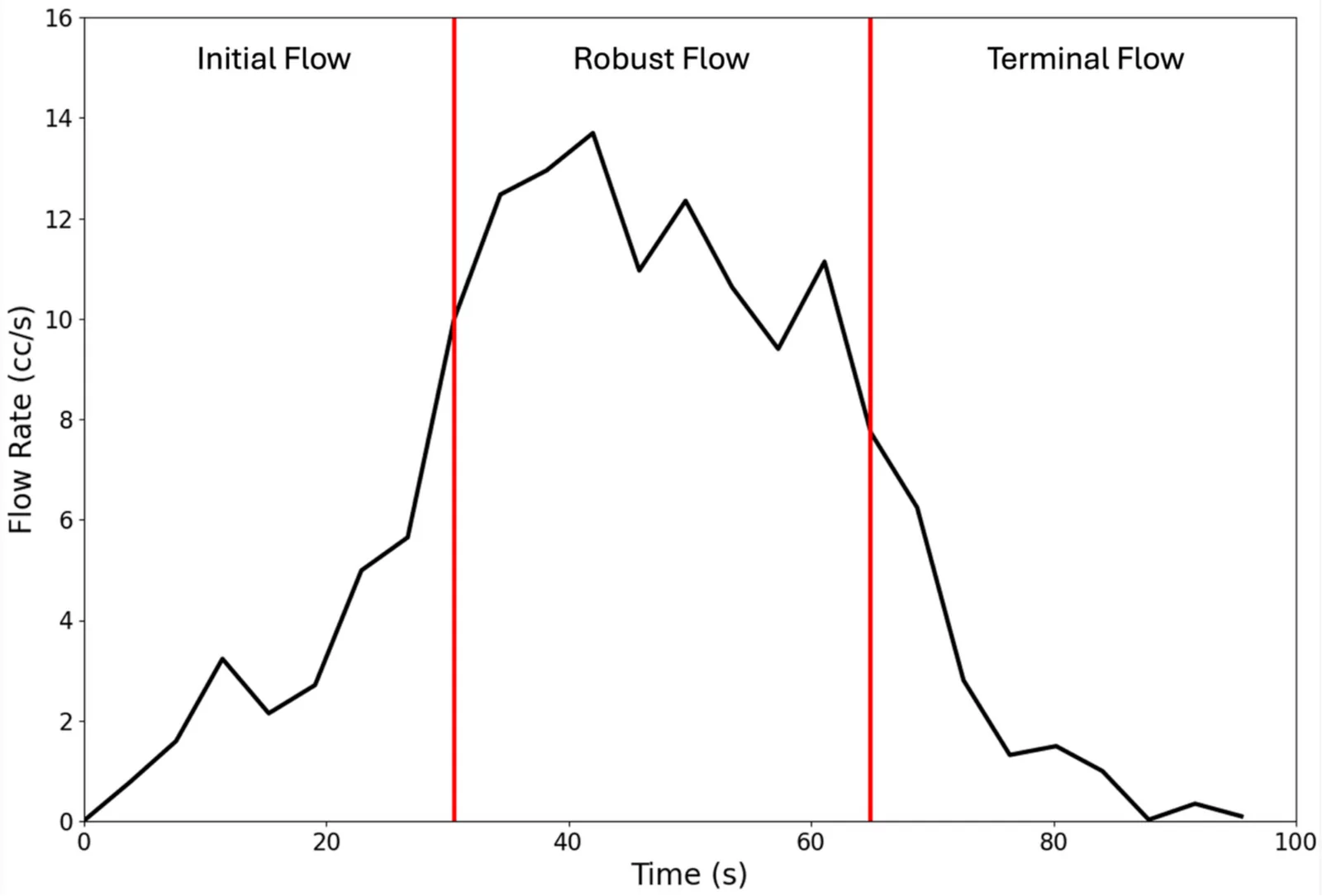
Calculated urine flow rate from change in bladder volume. The vertical red lines demarcate initial flow, robust flow, and terminal flow (left to right)

The performed dynamic imaging sequence of the single subject’s void in supine position yielded high fidelity, full field view oblique sagittal images of the urethra with appropriate contrast mixing (Fig. 2). The subject had a urethral length of 22.22 cm with urethral segment lengths as follows, prostatic: 3.25 cm, membranous: 1.17 cm, Bulbous: 6.47 cm, Penile: 11.33 cm. Flow rate was calculated from bladder volume change over time, resulting in an average flow rate between volumetric images collected every 3.82 s. Overall the subject had a maximum flow rate of 13.7 cc/s and an average flow rate of 5.6 cc/s, a urine flow curve is plotted over time in Fig. 4. The subject voided 532 cc in 95.5 s with a post void residual of 10 cc. For purposes of analysis, flow was divided into three sections: initial flow, robust flow, and terminal flow, with robust flow being when the flow rate was greater than 7 cc/s (30.56–64.94 s). The urethra was not completely visualized from 84 to 95.5 s due to the low urine volume being voided at that time.

Intra-reader variability studies were conducted using two trained readers yielding average urethral diameter variations for each flow section and overall bladder segmentation variation. The average variation in bladder segmentations, used to calculate flow rate, was between 1 and 8% throughout the voiding effort. Although the flow rate calculated from bladder volume change does not have the same temporal resolution as standard uroflowmetry, it provides time-matched biomechanic changes of the entire LUT while providing an average flow rate between two time-steps. The average urethral diameter variation between readers for initial, robust, and terminal flow is 0.12, 0.16 and 0.16 mm, respectively. Additionally, an inter-reader variability study was performed by one of the readers, yielding average urethral variations in all sections of flow. The average urethral diameter variation for initial, robust and terminal flow is 0.09, 0.15 and 0.11 mm, respectively. All variations in urethral diameters represent average errors between 3 and 5% of the smallest urethral diameter.

**Fig. 5:**
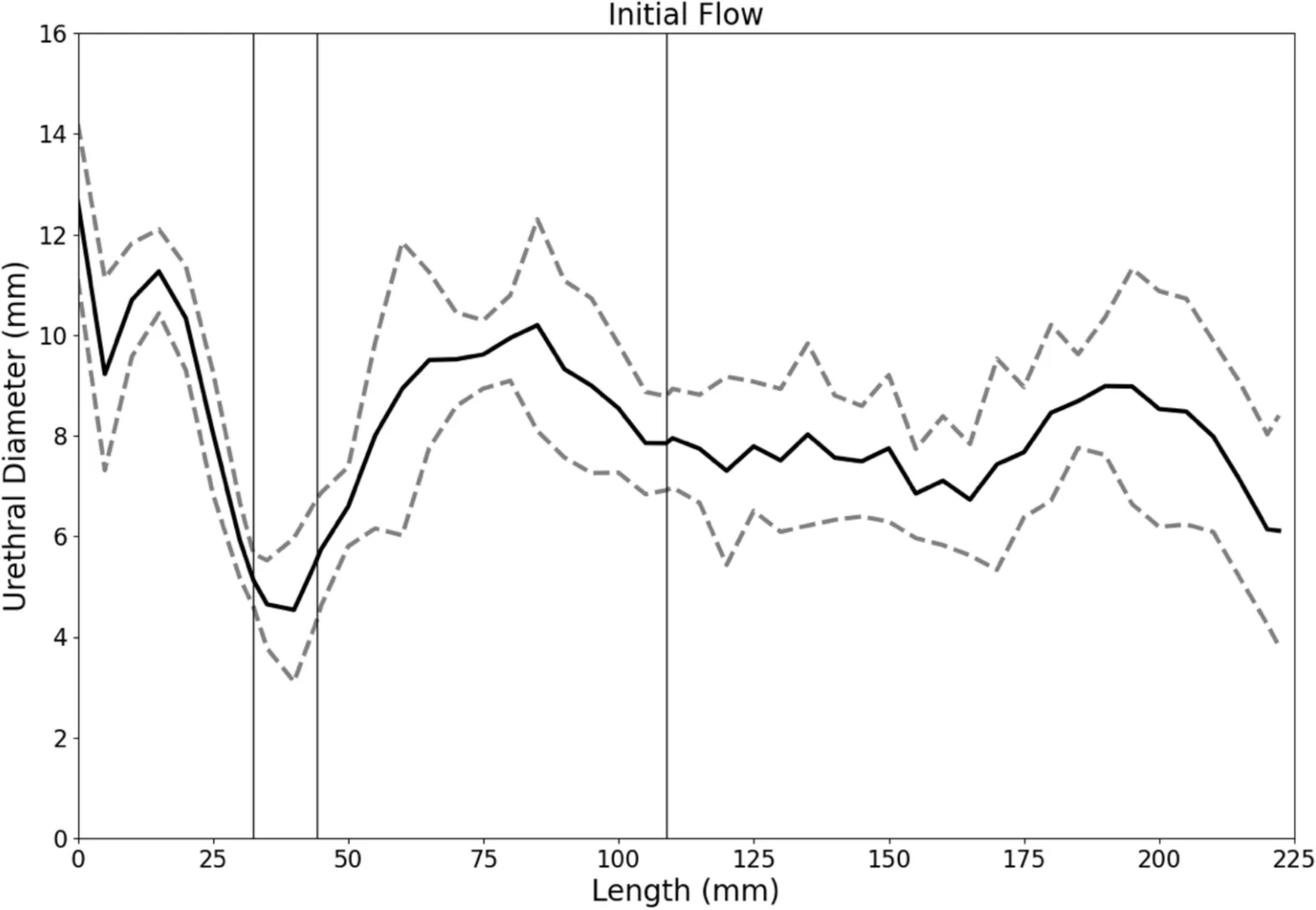
Average urethra diameter ± standard error (solid and dashed lines, respectively) over the length of the urethra were determined for initial flow from 7 time-steps (0–26.74 s). Vertical grey lines demarcate the prostatic urethra (0–32.5 mm), membranous urethra (32.5–44.2 mm), bulbous urethra (44.2–108.9 mm) and penile urethra (108.9–222 mm)

**Fig. 6:**
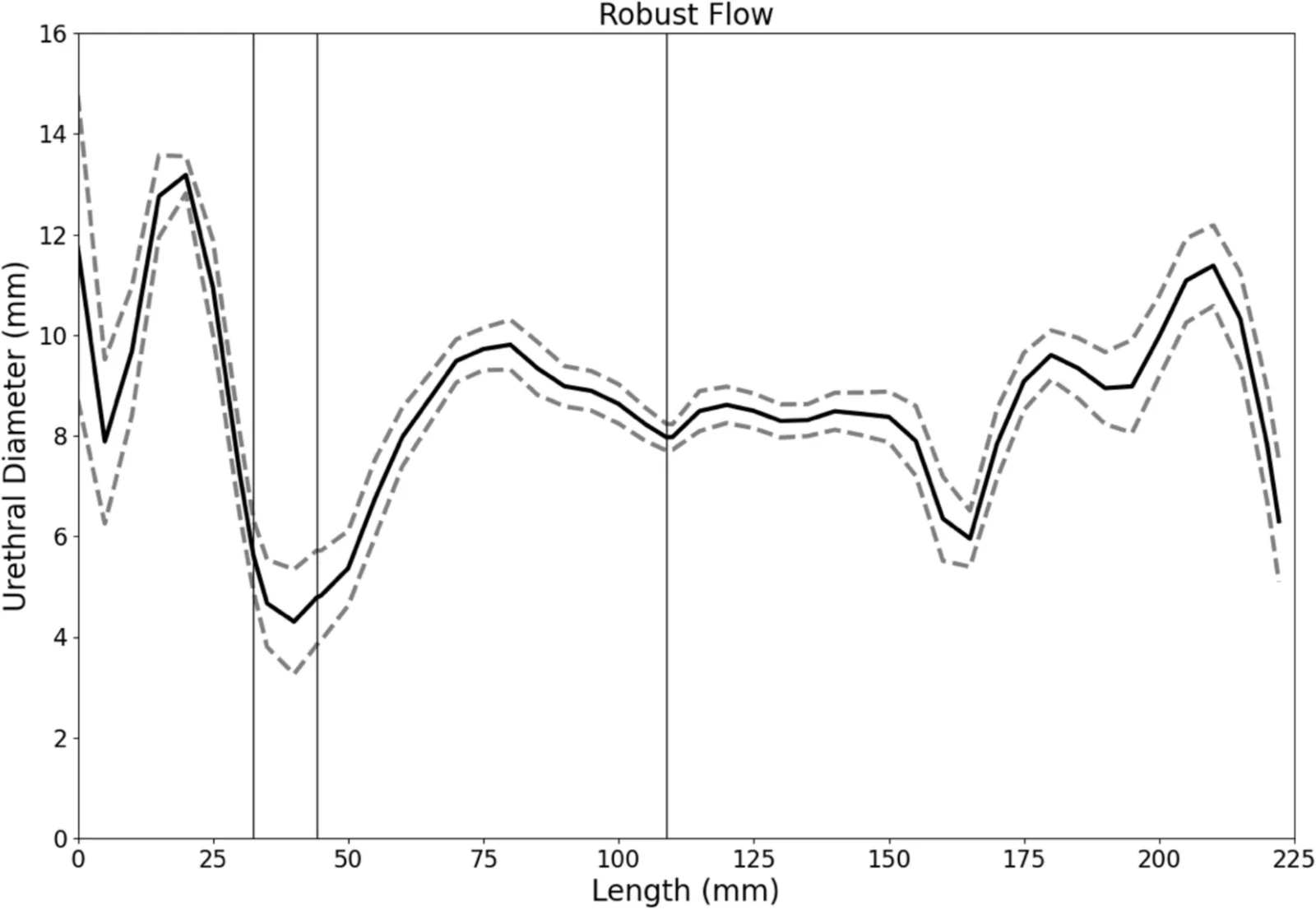
Average urethra diameter ± standard error (solid and dashed lines, respectively) over the length of the urethra were determined for robust flow from 11 time-steps (30.56–64.94 s). Vertical grey lines demarcate the prostatic urethra (0–32.5 mm), membranous urethra (32.5–44.2 mm), bulbous urethra (44.2–108.9 mm) and penile urethra (108.9–222 mm)

**Fig. 7:**
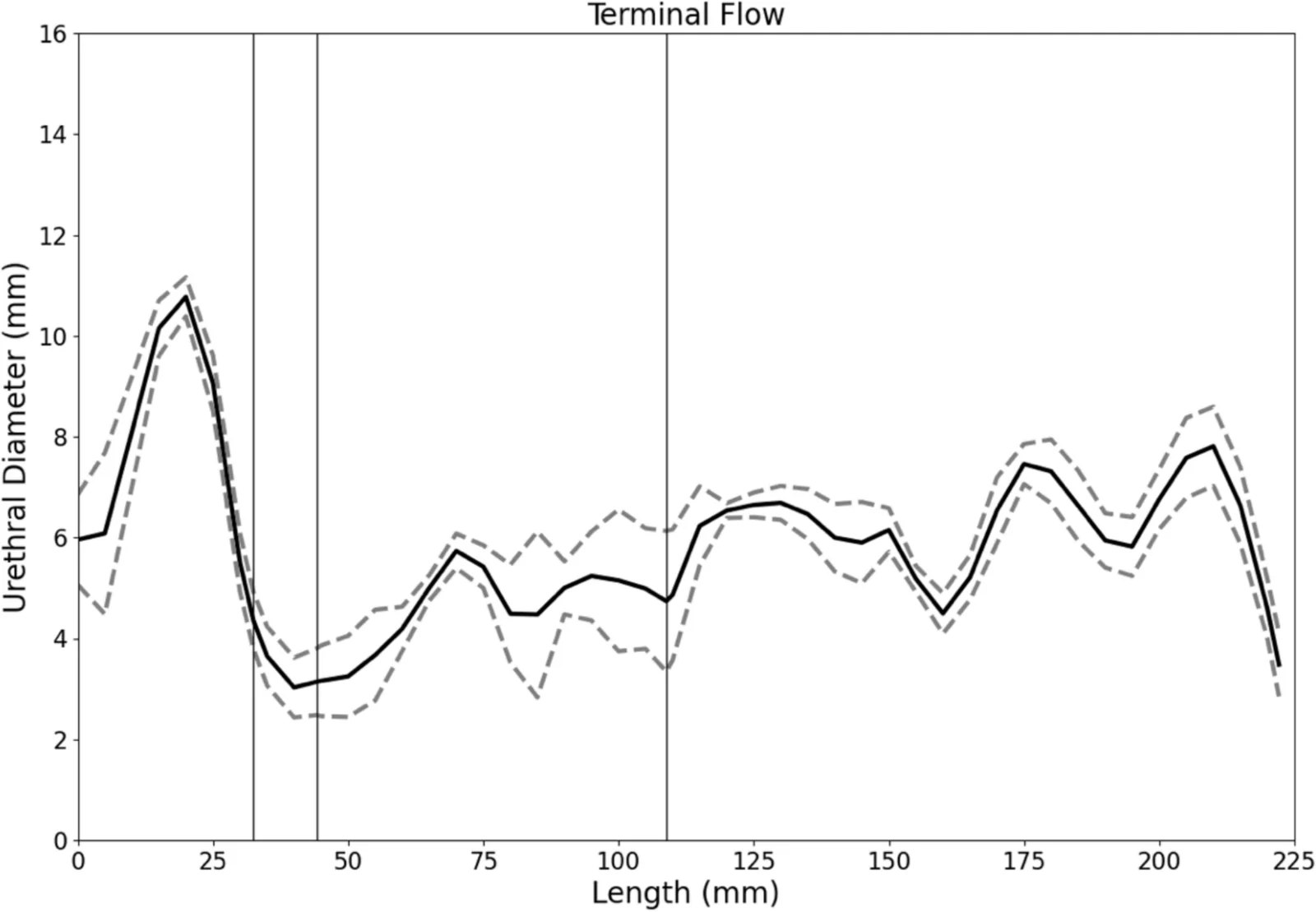
Average urethra diameter ± standard error (solid and dashed lines, respectively) over the length of the urethra were determined for terminal flow from 4 time-steps (68.76 s–84.04 s). Vertical grey lines demarcate the prostatic urethra (0–32.5 mm), membranous urethra (32.5–44.2 mm), bulbous urethra (44.2–108.9 mm) and penile urethra (108.9–222 mm)

The average urethral diameters and standard deviation were determined at each point along the length of the urethra during the periods of initial flow, robust flow, and terminal flow (Figs. 5, 6 and 7). Four observations are worth noting. First, there is marked variation in diameter in the bulbous and penile urethra during initial and terminal flow. This likely reflects the dependence of diameter of these compliant and capacious segments on flow rate, which varies considerably during initial and terminal flow. Second, the narrow point in the penile urethra evident during robust flow corresponds to a bend in the penis at proximal edge of the condom catheter used in this study. Third, the membranous urethra is the narrowest segment of the urethra in all three voiding periods. And fourth, both the proximal prostatic urethra and the membranous urethra exhibit relatively greater variation in diameter than the distal prostatic urethra during all three voiding periods and notably less than variation in diameter of the bulbous and penile urethra during robust flow.

### Step by step analysis

In the paragraphs below, this paper describes the quantitative variation in diameter of the different urethral segments through time, elucidating time dependent variations that are not captured by the time averaged values during the three periods shown in Fig. 5, 6 and 7. For clarity, the changes in the posterior urethra and anterior urethra are addressed separately.

#### Posterior urethra

Prior to the onset of urine flow and entry of contrast into the urethra, the urethral lumen cannot be imaged. At *t* = 0 s images show the bladder neck is completely closed. At the earliest imaged timepoint (3.82 s), the bladder neck was opened to a diameter of 15 mm and it remained open throughout voiding to an equal or greater diameter than any segment of the urethra (data not shown). Throughout the voiding effort, the most generous part of the prostatic lumen was around the seminal colliculus, with the proximal and distal parts of the prostatic urethra more narrowed. Regional changes within the prostatic urethra will be described using the form “proximal-seminal colliculus-distal”. At the earliest timepoint of flow, the prostatic urethra measured 13–12–5 mm. The narrowest point of the membranous urethra was 2 mm. At the second imaged timepoint (7.64 s) there was a decrease in the diameter of the prostatic urethra (8–10–5 mm) and an increase in the diameter of the membranous urethra (5 mm). From the third imaged timepoint (11.46 s) to near the end or robust flow (57.30 s), the urethral diameters remain relatively constant. While the prostatic urethral diameter exhibited little change, the narrowest diameter of the membranous urethra progressively decreased from 5 to 3 mm. During the terminal stage of voiding the diameter of the prostatic urethra diminished (5–11–5 mm) while the diameter of the membranous urethra remained constant (3 mm). With cessation of flow (87.86 s), the bladder neck and membranous urethra are closed while some remains in the prostatic urethra. At the next timepoint (91.68 s) urine remained in the prostatic urethra, however, at the next timepoint (95.5 s) urine is no longer present in the prostatic urethra but is still evident in the penile urethra.

#### Anterior urethra

The anterior urethra exhibited a consistent luminal diameter of approximately 8 mm throughout most of the voiding effort, decreasing to approximately 6 mm in the terminal phase. There was noted to be a focal narrowing in the mid-penile urethra and increased diameter in the distal penile urethra. As noted above, the narrowing corresponds to the proximal end of the condom catheter while the increased diameter at the distal tip appears to correlate with the larger diameter fossa navicularis.

### Data analysis

#### Average urethral diameters

**Fig. 8:**
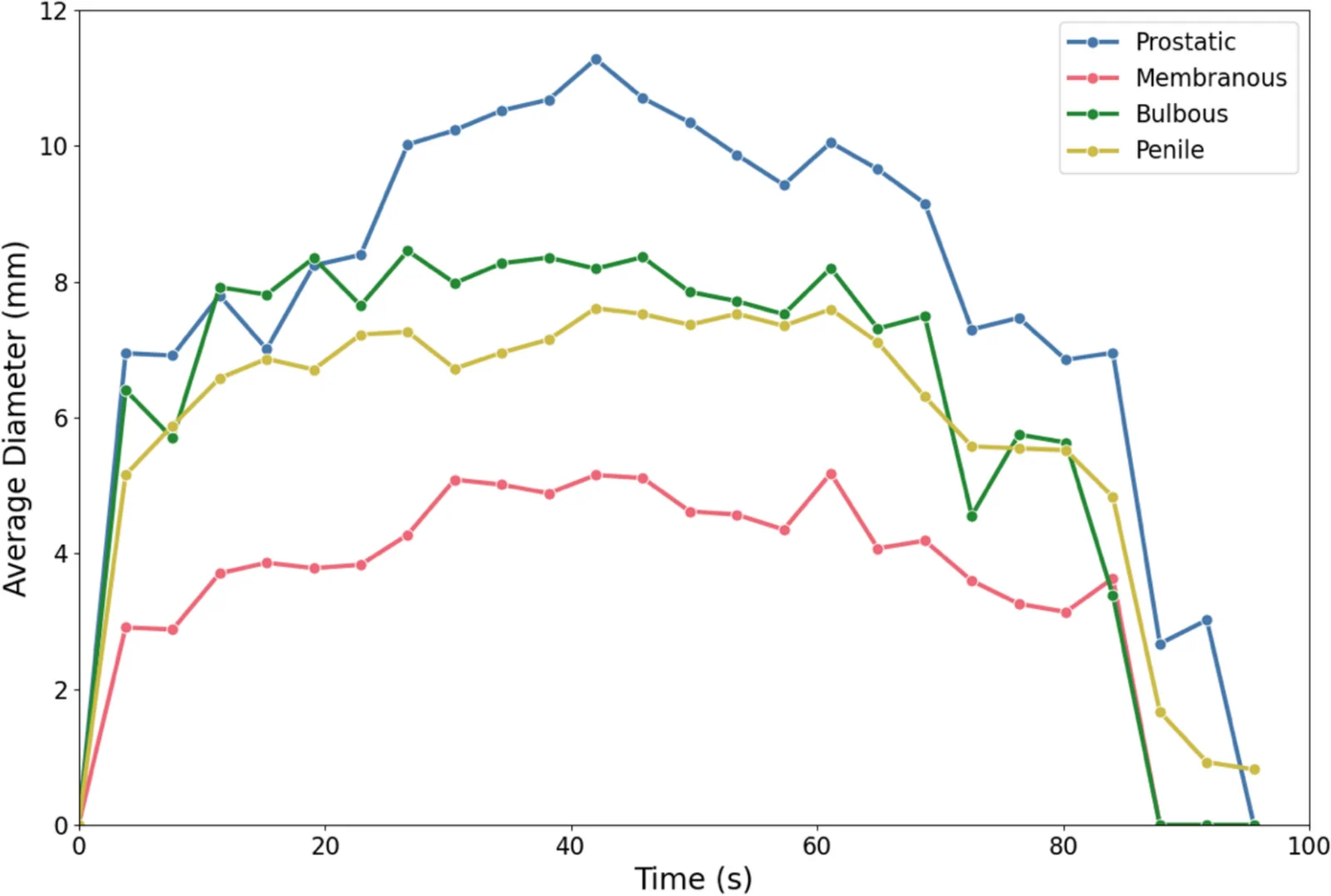
Urethral diameter divided into the four urethral segments (prostatic-blue, membranous-orange, bulbous-green, & penile-yellow) over time

The average diameter for each urethral segment was calculated by averaging each measurement along the urethral segment for each time step. These are then plotted for each of the four urethral segments (Fig. 8). This figure clearly illustrates that the membranous urethra exhibits the smallest diameter throughout the void.

#### Urethral resistance

**Fig. 9:**
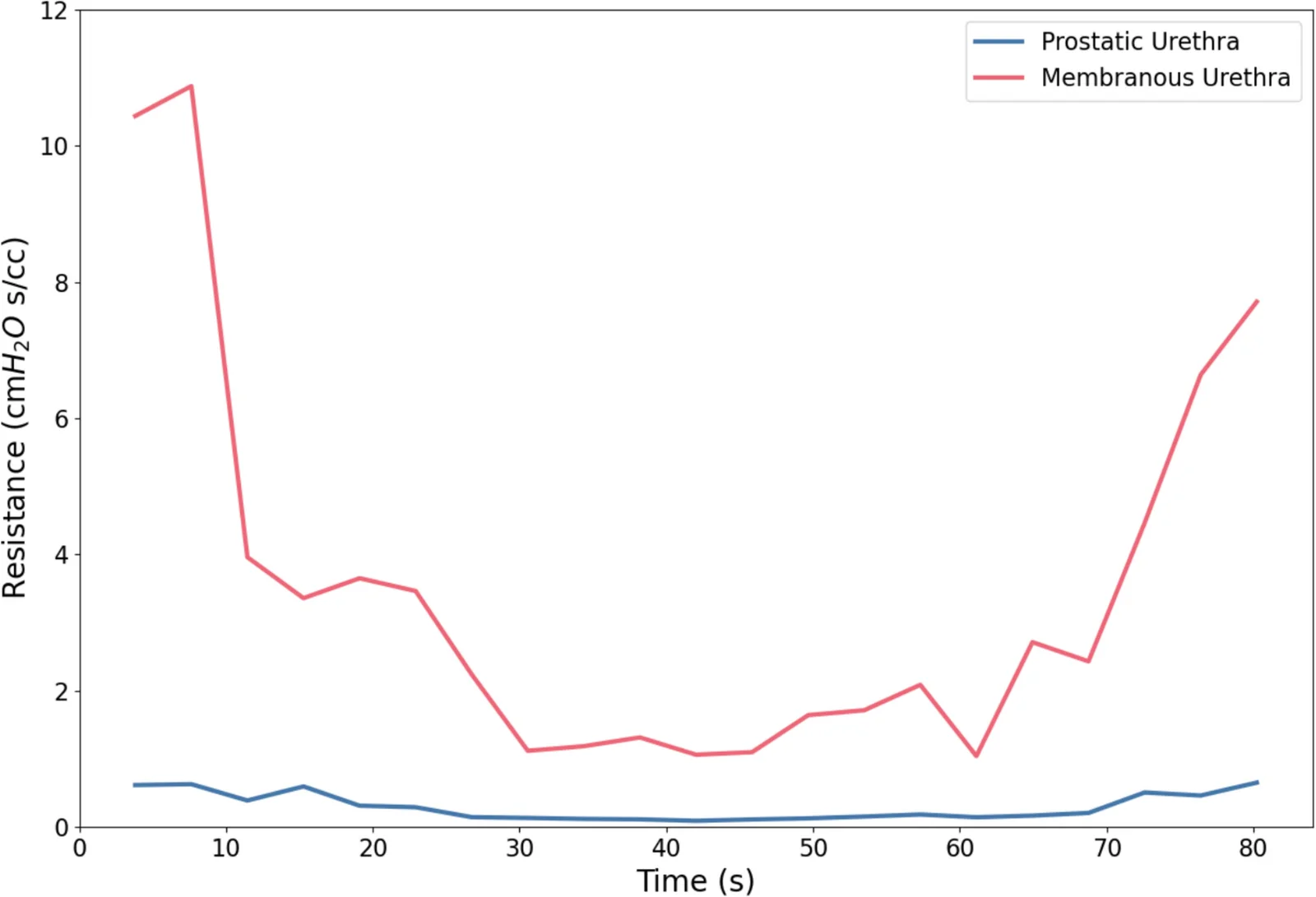
Prostatic and membranous urethral idealized instantaneous resistance plotted over time

Previous studies have reported low pressures and high compliance across the anterior urethra [20, 21, 23, 24]. Given the large diameters of the bulbous and penile urethra, as shown in Fig. 8, only the posterior urethra was considered for flow regulating resistances. While the membranous urethra exhibited the smallest diameter and might be expected to offer the greatest resistance, the prostatic urethra though having a larger diameter is significantly longer. To directly compare their relative resistance to flow by these two segments, idealized instantaneous resistances were calculated of the two segments and plotted them over time (Fig. 9). This indicates that the membranous urethra is the site of greatest resistance to flow throughout the voiding effort. Indeed, it is worth noting that the transient decrease in the membranous urethral resistance at 61.12 s corresponds to a transient increase in flow (Fig. 4).

**Fig. 10:**
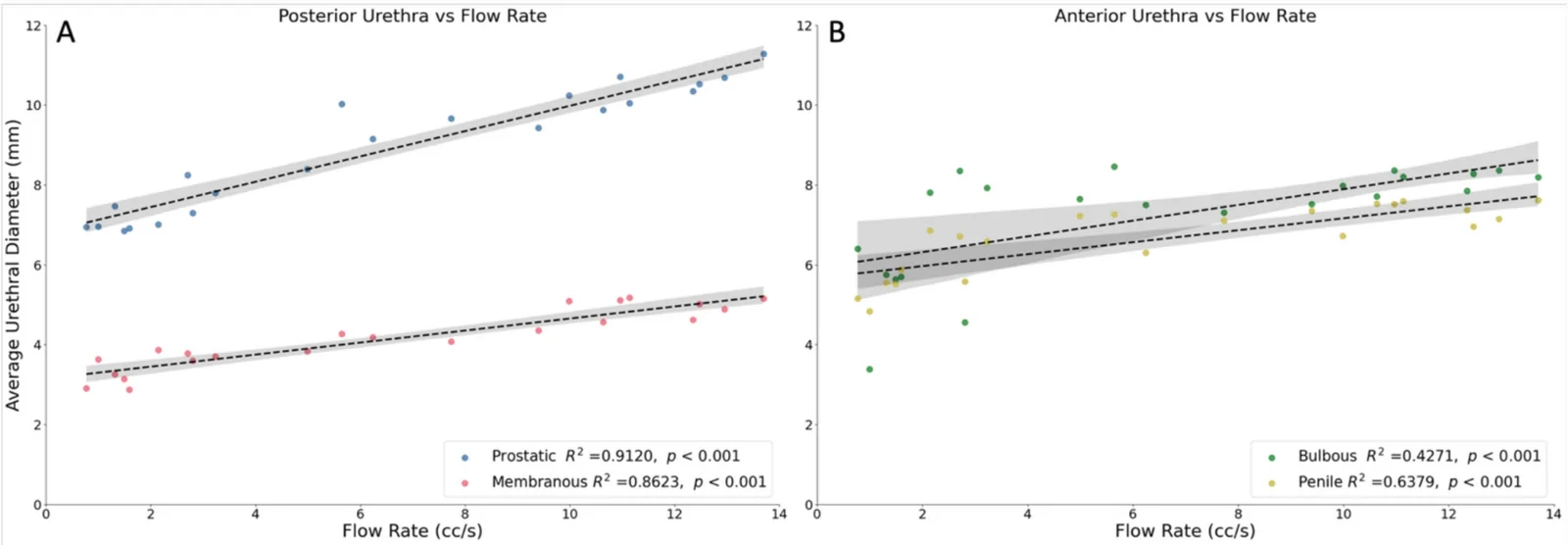
Average urethral diameters correlated with flow rate. **A** Prostatic and membranous urethra, **B** bulbous and penile urethra. Each graph shows *R*^2^ and *p* values for a 95% confidence interval, for each segment in the legend

The correlations of average diameter with flow rate were performed for each of the urethral segments (Fig. 10) using data from all the time-steps. The average diameters of the prostatic and membranous urethra showed a strong positive correlation with flow rate, *R*^2^ = 0.91 and 0.86, respectively. The diameters of the bulbous and penile urethra showed only a weak correlation (*R*^2^ 0.42 and 0.64, respectively). The low correlation of flow rate with the penile urethra might be an effect of the diameter restriction imposed by the condom catheter.

## Conclusions

This single-subject study demonstrates the feasibility and utility of using dynamic MRI to obtain dynamic, patient-specific images of the urethra during voiding. This protocol provides high resolution, time resolved images with multiplanar visualization of the entire urethral lumen and the entire LUT and has been validated *in-vivo* [10, 11] and extensively validated *in-vitro* [12]. Dynamically evaluating the urethra in supine position using a condom catheter could potentially induce anatomical changes in anterior urethra. Previous studies have demonstrated modest effects on quantitative measures of voiding pressure and urine flow [26, 27]. Further studies focusing on the anatomical changes of the urethra in voiding men should be performed to elucidate these differences. The obtained images enabled detailed segmentation and 3D renderings of the urethra from which urethral diameters and calculated idealized instantaneous resistances were obtained. Our results detailed distinct diameter changes during voiding. Analysis of diameters throughout the void revealed more variation in the initial flow and terminal flow than during robust flow. The most notable observation is that the membranous urethra exhibited the narrowest lumen and greatest resistance to flow. The prostatic urethra maintained a relatively generous lumen and low resistance throughout the void. These observations are consistent with previous studies performed with UDS, fluoroscopy and micturition urethral press profilometry [20, 21].

Correlation of urethral diameters with flow showed strong associations with changes in both the prostatic and membranous urethral diameters in this subject. Previous studies in normal young men have shown that the pressure in the prostatic urethra is isobaric to bladder with a significant pressure drop across the membranous urethra [20, 21, 25] and our studies of resistance are consistent with this. The results from this study are not to be taken as characterization of the normal urethra but as a demonstration of this methodology. A study with a statistically appropriate cohort size and multiple age ranges will be needed to further characterize urethral biomechanics during voiding.

This study expands recent advances in dynamic MRI for the characterization of bladder biomechanics [10–12] to the urethra. Results shown here reveal precise and dynamic visualization, and quantification of urethral geometry and flow rate during voiding. Dynamic quantification of anatomical changes of the urethra during voiding provide invaluable information to enhance the recently developed uro-dynamic MRI-based computational fluid dynamics method [28]. Future studies using these methodologies have the potential to increase understanding of the biomechanics of normal voiding and allow new insights into the mechanisms of voiding dysfunction.

## Data Availability

The participants of this study did not give written consent for their data to be shared publicly, so due to the sensitive nature of the research supporting data is not available.
